# Systematic RNA-interference in primary human monocyte-derived macrophages: A high-throughput platform to study foam cell formation

**DOI:** 10.1038/s41598-018-28790-3

**Published:** 2018-07-12

**Authors:** Gabriele Domschke, Fabian Linden, Lukas Pawig, Anna Hafner, Mohammadreza Akhavanpoor, Jürgen Reymann, Andreas O. Doesch, Christian Erbel, Christian Weber, Hugo A. Katus, Heidi Noels, Holger Erfle, Christian A. Gleissner, Heiko Runz

**Affiliations:** 1 Institute of Human Genetics, University of Heidelberg, 69120 Heidelberg, Germany; 2 Department of Cardiology, University of Heidelberg, 69120 Heidelberg, Germany; 3 DZHK (German Centre for Cardiovascular Research), Partner Site Heidelberg, 69120 Heidelberg, Germany; 4 Institute for Molecular Cardiovascular Research, RWTH Aachen University, University Clinic, 52074 Aachen, Germany; 5 BioQuant, Heidelberg University, 69120 Heidelberg, Germany; 6 Institute for Cardiovascular Prevention, LMU Munich and German Centre for Cardiovascular Research (DZHK), partner site Munich Heart Alliance, Munich, Germany; 7 Cardiovascular Research Institute Maastricht (CARIM), Maastricht University, Maastricht, The Netherlands; 8 Present Address: Biogen, Inc., Cambridge, MA USA

## Abstract

Macrophage-derived foam cells are key regulators of atherogenesis. They accumulate in atherosclerotic plaques and support inflammatory processes by producing cytokines and chemokines. Identifying factors that regulate macrophage lipid uptake may reveal therapeutic targets for coronary artery disease (CAD). Here, we establish a high-throughput screening workflow to systematically identify genes that impact the uptake of DiI-labeled low-density lipoprotein (LDL) into monocyte-derived primary human macrophages. For this, monocytes isolated from peripheral blood were seeded onto 384-well plates, solid-phase transfected with siRNAs, differentiated *in vitro* into macrophages, and LDL-uptake per cell was measured by automated microscopy and quantitative image analysis. We applied this workflow to study how silencing of 89 genes impacts LDL-uptake into cells from 16 patients with CAD and 16 age-matched controls. Silencing of four novel genes (*APOC1*, *CMTM6*, *FABP4*, *WBP5*) reduced macrophage LDL-uptake. Additionally, knockdown of the chemokine receptor *CXCR4* reduced LDL-uptake, most likely through a G-protein coupled mechanism that involves the CXCR4 ligand macrophage-induced factor (MIF), but is independent of CXCL12. We introduce a high-throughput strategy to systematically study gene function directly in primary CAD-patient cells. Our results propose a function for the MIF/CXCR4 signaling pathway, as well as several novel candidate genes impacting lipid uptake into human macrophages.

## Introduction

Coronary artery disease (CAD) is an inflammatory disease of the arterial vessel wall and underlies myocardial infarction as the most common cause of death worldwide^1^. During atherogenesis, vascular microlesions increase endothelial permeability for low-density lipoprotein (LDL)-cholesterol. LDL retained in the arterial vessel wall can be oxidized, thereby enhancing migration of monocytes from the blood into the subendothelial space. Uptake of LDL by monocyte-derived macrophages contributes to foam cell formation and secretion of cytokines and chemokines. This attracts further immune and vascular smooth muscle cells, leading to sustained inflammation and a progression of fatty streaks to irreversible alterations in the arterial wall, which constitutes the atherosclerotic plaque. Over time, this lesion may become instable, rupture, and lead to platelet thrombus formation and ultimately myocardial infarction^2,3^.

Foam cells are the consequence of an imbalanced lipid uptake versus efflux in macrophages. Lipid uptake from native or modified LDL is mediated by the LDL-receptor, or scavenger receptors such as CD36 and SR-A, respectively, with minor fractions entering cells through additional mechanisms^4,5^. The transformation of monocyte-derived macrophages into foam cells is a key event during plaque formation: first, foam cells induce inflammation through release of a variety of cytokines and chemoattractants; second, foam cells maintain inflammation in the plaque through undergoing apoptosis, which triggers further pro-inflammatory processes; and third, apoptotic foam cells constitute the physical center of the plaque that critically impacts plaque progression, destabilization, and rupture^6^. Thus, pharmacological strategies that suppress foam cell formation are highly sought after for the prevention or treatment of CAD^7,8^.

Here, we establish a high-throughput screening workflow to systematically study the impact of siRNA-induced loss-of-function of candidate genes on LDL-uptake into monocyte-derived macrophages from CAD patients and controls. We propose several novel candidate genes and the MIF/CXCR4 signaling pathway as likely functional regulators of foam cell formation in humans.

## Results

### Quantifying the functional impact of candidate gene knock-down on foam cell formation from primary human monocyte-derived macrophages

**Figure 1:**
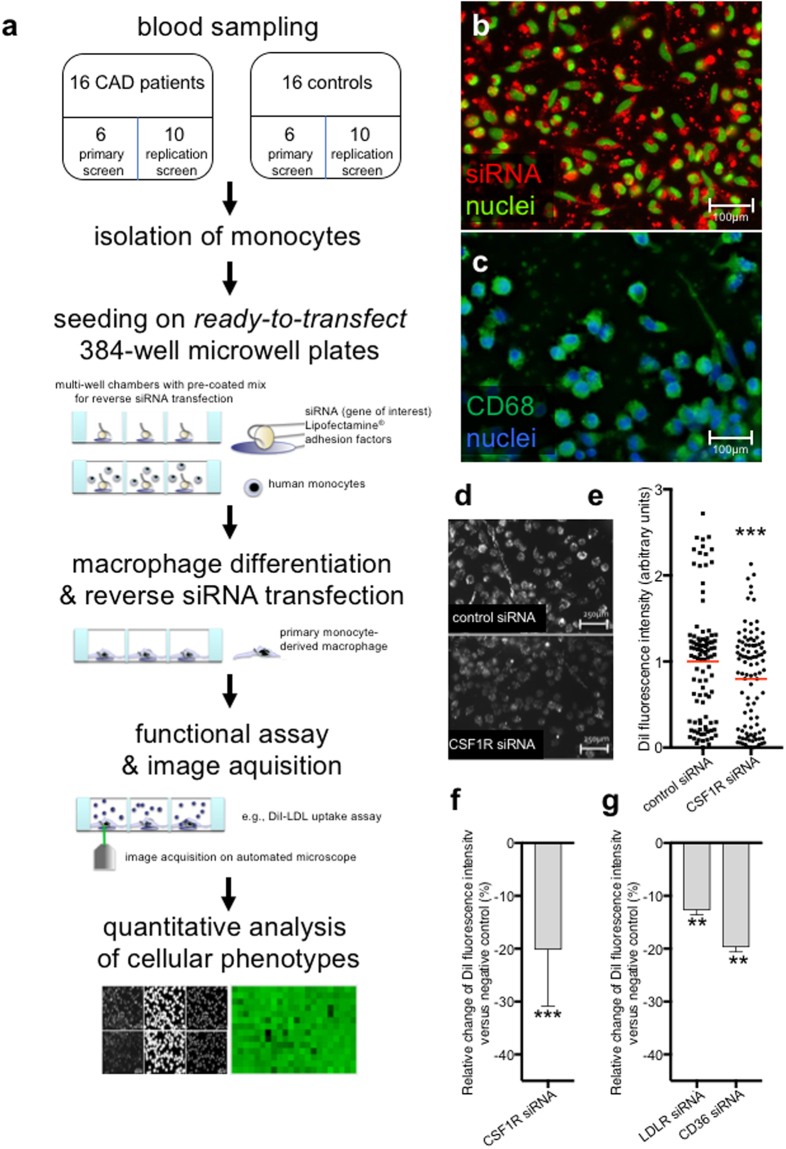
Microscope-based screening platform for solid-phase siRNA-transfection and functional analyses in primary human monocyte-derived macrophages. (**a**) Workflow illustrating the experimental setup: Blood sampling, monocyte isolation and seeding on siRNA-coated plates, monocyte-macrophage differentiation and solid-phase siRNA transfection, functional assays, image acquisition and analysis. (**b**,**c**) Representative images reflecting siRNA transfection efficiency as monitored with Cy3-labelled non-silencing control-siRNA (b, red) and macrophage marker CD68 (c, green) in monocyte-derived macrophages three days after siRNA transfection. (**d**) Representative images of DiI-LDL signals in cells transfected with either control siRNA or siRNA against *CSF1R*. (**e**,**f**) Relative DiI signal intensities in macrophages from 6 healthy individuals treated with control or CSF1R-siRNA as measured through quantitative image analysis. Each dot in (**e**) indicates the mean cellular DiI signal intensity per 1 frame from 15 technical replicates per individual (i.e., independent wells of a plate). (**f**) Shows change in DiI signal relative to negative control in percent (±s.d.). (**g**) Mean±s.d. relative change in DiI signal compared to negative control in percent for siRNAs against *LDLR* and *CD36*. Macrophages from 6 healthy individuals were used with 12 technical replicates per individual. P values were calculated with Bonferroni corrected unpaired two-tailed t-test. **P < 0.001; ***P < 0.0001.

To systematically study the impact of multiple genes on foam cell formation in parallel, we established a workflow that enabled parallelized RNA interference (RNAi), automated microscopic image acquisition, and quantitative phenotypic analyses from primary blood monocyte-derived macrophages at the level of individual cells. To this end, we optimized technology for reverse siRNA transfection from “ready to transfect” multi-well plates^9^ for being suitable to study primary human blood-derived cells (Fig. 1a and Methods). For solid-phase reverse siRNA transfection, CD14^+^ monocytes were isolated from peripheral blood of healthy or diseased donors, seeded at a density of 20,000–25,000 cells/well on 384-well plates, and differentiated with macrophage colony-stimulating factor (MCSF) prior to realization of cell-based assays, automated microscopy and quantitative image analysis. A fluorescent-labeled siRNA confirmed that through this strategy nearly 100% of seeded cells could be successfully siRNA-transfected (Fig. 1b). Differentiation from monocytes towards macrophages followed a protocol we and others have described previously^10,11^ and was monitored with an antibody against CD68 (Fig. 1c). Out of four assays we had described earlier as suited for monitoring lipid accumulation in cells under a high-throughput setting^12,13^ and that are in principle amenable also to macrophages [including lipid droplet formation (using Oil Red O); estimation of cellular cholesterol content (using filipin); and determining cellular uptake of either native or oxidized fluorescent-labeled low-density lipoprotein (DiI-LDL and DiI-oxLDL) (Fig. S1)], we chose to optimize measuring DiI-LDL uptake for a proof-of-concept high-throughput screening study in primary patient-derived macrophages. A significant reduction in LDL-uptake relative to controls could be quantified when cells were transfected with siRNAs targeting positive controls such as the LDL-receptor (*LDLR*), the cholesterol scavenger receptor *CD36*, or the MCSF receptor (*CSF1R*)^5^ (Fig. 1d–g).

### RNAi-screening of genes induced during foam cell formation identifies novel regulators of macrophage LDL-uptake

With the aim to identify novel regulators of LDL-uptake into monocyte-derived macrophages that might serve as therapeutic targets for CAD, we applied our platform to conduct a systematic RNAi-screen. We focused on a set of 89 genes which have been described earlier to be human macrophage-expressed genes that change expression in response to lipid loading^10,14^ (Table S1; see Methods for details on candidate gene selection). Primary blood-derived monocytes were obtained from 16 CAD patients and 16 healthy control individuals. CAD patients tended to be older and more likely to be hypertensive or diabetic, yet no differences were seen regarding a diagnosis of hyperlipidemia, smoking, or family history for cardiovascular disease (see Table S2 for detailed participant characteristics). A primary screen was conducted in cells from 6 patients/controls, a replication screen in cells from 10 patients/controls. Cells were solid-phase reverse siRNA transfected, differentiated towards macrophages, assayed for DiI-LDL uptake, and image acquisition and quantitative analysis was performed.

**Figure 2:**
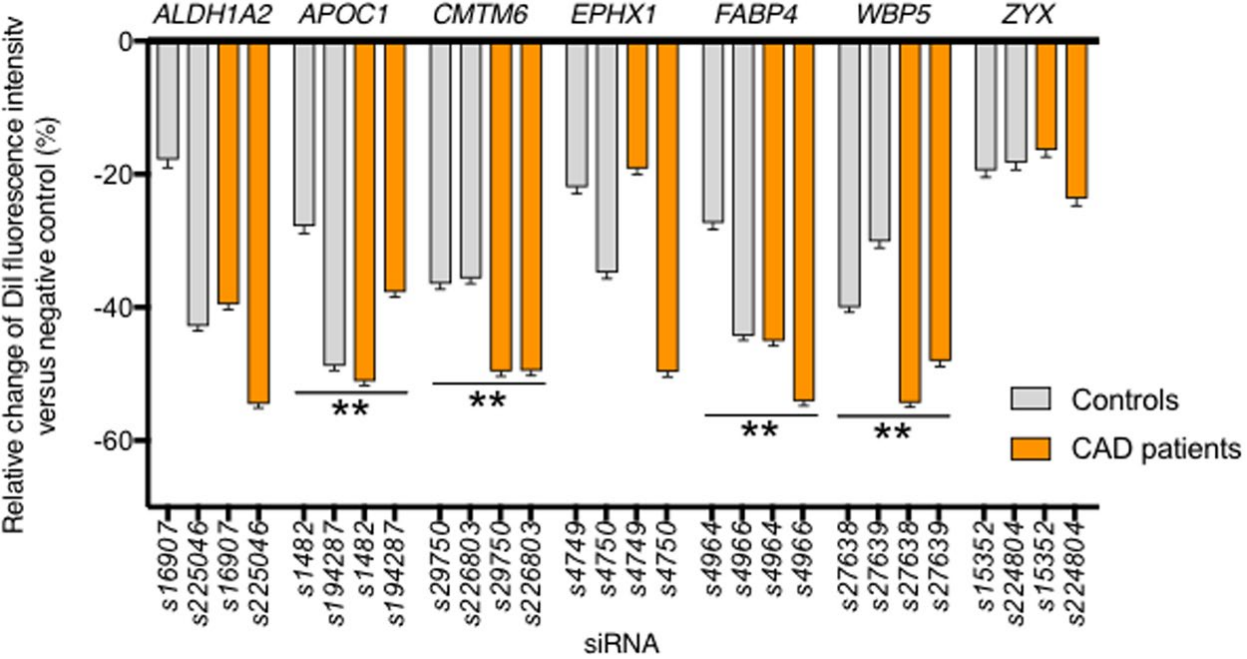
Knockdown of *APOC1*, *CMTM6*, *FABP4* and *WBP5* reduces DiI-LDL uptake into primary human monocyte-derived macrophages. Bars reflect mean ± S.D relative change in cellular DiI-LDL fluorescence signal intensities as determined from cultured primary human monocyte-derived macrophages during the replication screen. Cells were solid-phase transfected with siRNAs against indicated genes (2 independent siRNAs/gene), and DiI-LDL uptake was measured relative to control siRNA treated cells with 1.0 being the mean intensity of all negative control wells for each plate. Shown are results from cells of 10 healthy individuals (grey) and 10 patients with coronary artery disease (orange). To determine significance, deviation values (Dev; see Methods) were calculated for each siRNA tested in each individual relative to the mean intensity of respective negative control wells, and the median deviation was calculated for the control group (Md(C)), the patient group (Md(P)), and the combination of both groups (Md(C + P)) separately. Finally, the mean median deviation shown was calculated from the median deviation of results from three technical replicates per individual (i.e, three independent parallel analyses of cells isolated from the same blood draw). **Dev <−1.0 for at least 2 siRNAs in 1 group (controls, patients, controls + patients). For full siRNA screening results from primary and replication screens, see Supplemental Data.

For seven of the 89 genes studied, siRNA knockdown caused significant changes in DiI-LDL uptake during the primary screen (Table 1; see Supplemental Data for full screening results). Specifically, knockdown of five genes (*ALDH1A2*, *APOC1*, *CMTM6*, *FABP4* and *WBP5*) was found to significantly downregulate DiI-LDL uptake, whereas individual siRNAs against two genes (*EPHX1* and *ZYX*) appeared to increase DiI-LDL uptake into monocyte-derived macrophages. Of these seven putative novel effector genes, four genes, *APOC1*, *CMTM6*, *FABP4* and *WBP5*, could be confirmed with ≥2 siRNAs/gene as novel regulators of LDL-uptake into macrophages also in the independent replication screen (Table 1, Fig. 2, Fig. S2). Interestingly, knockdown of *WBP5* reduced LDL-uptake more strongly in macrophages derived from the CAD patients than in healthy controls (Supplemental Data).

**Table 1:** Genes identified as functional regulators of DiI-LDL uptake into cultured primary human monocyte-derived macrophages.

Gene	siRNA ID	Primary Screen			Replication Screen		
		Mean Md(C)	Mean Md(P)	Mean Md(C + P)	Mean Md(C)	Mean Md(P)	Mean Md(C + P)
ALDH1A2	s16908	0.9	0.3	0.6	n.a.	n.a.	n.a.
	s225046	−0.2	**−1**.**2**	−0.5	**−1**.**2**	**−1**.**8**	**−1**.**4**
	s16907	0.8	−0.9	−0.3	−0.8	−0.9	−0.8
APOC1**	s194287	**−1**.**4**	−0.6	**−1**.**2**	**−1**.**1**	**−1**.**5**	**−1**.**1**
	s1481	0.1	0.1	0.1	n.a.	n.a.	n.a.
	s1482	−0.5	−0.8	−0.7	−0.9	**−1**.**5**	**−1**.**0**
CMTM6**	s29750	**−1**.**0**	0.0	−0.2	**−1**.**0**	**−1**.**5**	**−1**.**2**
	s226803	−0.7	−0.6	−0.6	−0.9	**−1**.**6**	**−1**.**1**
	s29749	0.3	0.3	0.3	n.a.	n.a.	n.a.
EPHX1	s4750	−0.1	**1**.**5**	0.2	−0.9	**−1**.**6**	**−1**.**1**
	s4749	0.5	0.9	0.7	−0.6	−0.4	−0.5
	s4751	−0.1	−0.2	−0.2	n.a.	n.a.	n.a.
FABP4**	s4965	0.0	0.5	0.2	n.a.	n.a.	n.a.
	s4966	**−3**.**1**	**−2**.**6**	**−2**.**9**	**−1**.**2**	**−2**.**0**	**−1**.**3**
	s4964	−0.6	**−1**.**2**	**−1**.**0**	−0.7	**−1**.**3**	−0.9
WBP5**	s27638	−0.8	0.2	−0.3	**−1**.**1**	**−2**.**2**	**−1**.**4**
	s27637	−0.3	0.3	−0.1	n.a.	n.a.	n.a.
	s27639	**−1**.**1**	−0.4	−0.7	−0.9	**−1**.**9**	**−1**.**1**
ZYX	s15351	−0.1	0.2	−0.1	n.a.	n.a.	n.a.
	s15352	0.8	−0.1	0.1	−0.4	−0.3	−0.3
	s224804	**1**.**6**	0.6	0.8	−0.5	−0.5	−0.5

The impact on DiI-LDL uptake upon candidate gene knockdown was analyzed with 3 independent siRNAs/gene. SiRNAs meeting significance criteria (z-score/Deviation >|1|) are displayed in bold as mean medians of deviations (Md). Genes with ≥2 significant siRNAs validated in replication screen are highlighted by**. Negative values indicate reduction in DiI-LDL uptake relative to control-siRNA transfected cells. C, control individuals; P, coronary-artery disease patients; column Md(C + P) indicates means across patients and control individuals in this study. n.a., not analyzed.

### RNAi delineates a role for MIF/CXCR4 signaling during foam cell formation

**Figure 3:**
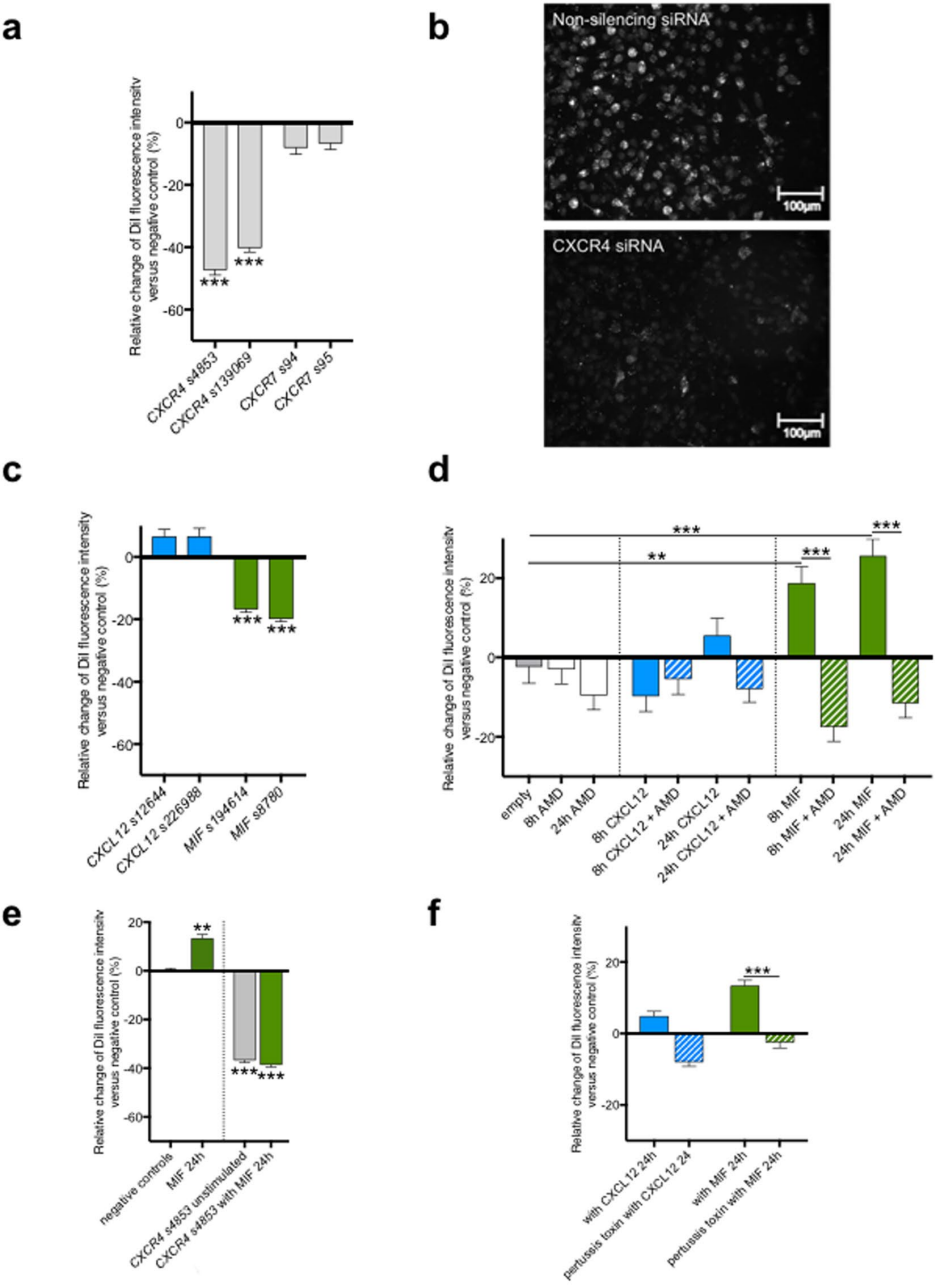
The MIF-CXCR4 signaling axis regulates DiI-LDL uptake into primary human monocyte-derived macrophages. (**a**) Relative change in DiI signal intensity compared to negative control (in percent) in macrophages treated with siRNAs against *CXCR4* or *CXCR7* (n = 5). (**b**) Representative images of DiI-LDL signal in macrophages treated with CXCR4- or control-siRNA. **(c)** Relative change in DiI signal intensity compared to negative control in per cent in macrophages treated with siRNAs against *CXCL12* or *MIF* (n = 3). (**d**) Relative change in DiI fluorescence intensity compared to negative control (untreated cells) after stimulation with either AMD3100 (AMD) alone for 8 or 24 hours, or CXCL12 (1 μg/ml) or MIF (1 μg/ml) for 8 or 24 hours in the absence or presence of AMD (n = 3). (**e**) Relative change in DiI fluorescence intensity compared to negative control siRNA after treatment with CXCR4-siRNA in the absence or the presence of MIF (1 μg/ml) for 24 hours. (**f**) Relative change in DiI fluorescence intensity compared to negative control (untreated cells) after stimulation with MIF (1 μg/ml) in the absence or presence of pertussis toxin (250 ng/ml) for 24 hours (n = 3). P-values were calculated with unpaired, two-tailed t-test followed by a post-hoc Dunn’s multiple comparison test. ***P < 0.0001.

In addition to screening for novel modifiers of foam cell formation, we further wanted to test whether systematic analyses in human monocyte-derived macrophages on our platform could help to shed light on known pathways of foam cell regulation. For this, we chose to investigate the roles of CXCR4 and CXCR7 in this process. Both proteins are chemokine receptors that previous studies, predominantly from mice, have proposed as relevant for lipid accumulation in macrophages, atherosclerosis and CAD, although the mechanisms remain unclear^15^. In our screening system, knockdown of *CXCR4* strongly reduced DiI-LDL uptake, while silencing of *CXCR7* did not impact this process (Fig. 3a,b). This suggests that, at least *in vitro*, the relative contribution of CXCR4 to foam cell formation from human monocyte-derived macrophages exceeds that of CXCR7.

In order to gain insights into the underlying mechanisms, we systematically studied the relevance of different components of the CXCR4 signaling pathway on DiI-LDL uptake. For this, we first silenced the CXCR4 ligands CXCL12 and MIF^16^. Silencing of *CXCL12* did not impact DiI-LDL uptake in our system. However, knockdown of *MIF* reduced DiI-LDL uptake into human monocyte-derived macrophages (Fig. 3c). This suggests that CXCR4 impacts foam cell formation via the MIF signaling axis rather than via CXCL12.

To further validate these findings, we incubated monocyte-derived macrophages with MIF and CXCL12 proteins for 8 and 24 hours, respectively, and measured DiI-LDL uptake. Consistent with the findings upon knockdown, addition of CXCL12 protein did not affect DiI-LDL uptake. Conversely, addition of MIF significantly stimulated LDL uptake in human monocyte-derived macrophages at both time points measured (Fig. 3d). To confirm that the impact on LDL-uptake was indeed conveyed by the interplay of MIF with CXCR4, we blocked interaction of MIF with CXCR4 using the compound AMD3100 (1-[4-(1,4,8,11-Tetrazacyclotetradec-1-ylmethyl)phenyl]methyl}-1,4,8,11-tetrazacyclo-tetradecan, Plerixafor), a clinically-approved specific inhibitor of ligand binding to CXCR4^17^. While AMD3100 did not significantly impact DiI-LDL uptake either alone or in combination with CXCL12, it completely reverted the MIF-induced increase in DiI-LDL uptake (Fig. 3d). Consistent with MIF modulating DiI-LDL uptake through CXCR4, silencing of *CXCR4* completely overruled upregulation of DiI-uptake by MIF protein (Fig. 3e). Since CXCR4 is a G-protein coupled receptor (GPCR), we speculated that CXCR4 might impact foam cell formation via a G-protein mediated mechanism. Indeed, incubation of human monocyte-derived macrophages with GTP-signaling inhibiting pertussis toxin for 24 hours fully abrogated the LDL-increasing effect of MIF on CXCR4 (Fig. 3f). Taken together, these results propose a role for the MIF/CXCR4 signaling pathway in the transformation of human monocyte-derived macrophages to foam cells. They further suggest that blocking MIF-CXCR4 interaction or GTP-mediated downstream signaling could be promising strategies to reduce lipid accumulation in macrophages as an important driver of atherosclerotic plaque formation.

## Discussion

Here we introduce a novel platform to systematically conduct RNAi-screens in human primary monocyte-derived macrophages. Our platform is based on the parallelized solid-phase reverse siRNA transfection of monocytes isolated from peripheral human blood, functional assays in monocyte-derived macrophages, automated microscopy, and quantitative image analysis. To demonstrate applicability of this platform, we conduct an RNAi-screen to identify regulators of macrophage LDL-uptake among 89 genes that were previously reported as differentially regulated in foam cells. We show that knockdown of *APOC1*, *CMTM6*, *FABP4* and *WBP5* reduces the uptake of fluorescent-labeled LDL into macrophages from 32 individuals (16 CAD patients and 16 healthy controls), suggesting that a reduced function of these genes could negatively impact foam cell formation. Furthermore, we substantiate evidence for CXCR4 as a critical regulator during atherogenesis by proposing a role for the MIF/CXCR4 signaling pathway on macrophage LDL-uptake that is potentially amenable to therapeutic intervention.

Macrophages constitute a critical cell type during development and destabilization of atherosclerotic plaques^5–8,18^. By internalizing lipids and transforming into foam cells, they promote the progression of an atherosclerotic lesion and initiate and maintain inflammatory processes. While de-differentiated human macrophage model cell lines such as THP-1 cells are now increasingly used in screening settings, most insights into macrophage biology and their role in disease are derived from animal models. Human primary monocyte-derived macrophages without further differentiation have, to the best of our knowledge, thus far not been analyzed in systematic RNAi-screens, although the opportunity to analyze primary cells derived directly from peripheral blood of patients may offer significant advantages.

In our study, we queried for genes that modulate LDL-uptake into monocyte-derived macrophages, and thus could potentially serve as therapeutic targets for enabling cardiovascular therapies. We followed a paradigm that we had successfully applied earlier^12,13^ and analyzed a set of genes that had previously been identified as differentially regulated at the transcriptional level during foam cell formation for an impact on the internalization of fluorescent-labelled LDL into cells, a critical constituent during foam cell formation from macrophages^5^. In two independent screens in cells from both, CAD patients and controls, we identified four genes as consistent modulators of macrophage DiI-LDL uptake: *APOC1*, *CMTM6*, *FABP4* and *WBP5*.

*APOC1* encodes apolipoprotein C-I, a protein with central functions in lipoprotein metabolism. *APOC1* is part of the *TOMM40/APOE/APOC1* gene cluster on chromosome 19q13 that has been associated through GWAS via rare and common alleles with plasma lipid levels and carotid intima media thickness among others^19,20^. Various studies have investigated the role of *APOC1* in the regulation of plasma lipid levels^21,22^, and elevated APOC1 plasma levels have been proposed as potentially aggravating CAD and myocardial infraction in mice and humans^23,24^. *APOC1* is highly expressed during monocyte-to-macrophage differentiation^25^ and modulates phospholipid and cholesterol efflux from macrophages^23^. That knockdown of *APOC1* modulates DiI-LDL uptake supports our earlier observations from HeLa cells where independent *APOC1* siRNAs also tended to lower LDL-internalization, although in our earlier study they had failed to meet our stringent significance thresholds^13^. Future studies will need to determine the exact mechanisms how APOC1 impacts macrophages.

Fatty acid binding protein-4 (*FABP4*) is a small cytoplasmic lipid chaperone that binds long-chain fatty acids and regulates lipid-induced macrophage endoplasmic reticulum (ER) stress^26,27^. Elevated FABP4 levels have been linked to a modestly higher risk of heart failure, and sudden cardiac death^28,29^, and a variant near *FABP4* was proposed to reduce the risk of cardiovascular incidents and increase plaque stability^30,31^. Genetic or chemical inhibition of FABP4 alleviates macrophage ER stress and protects against atherosclerosis in an animal model^27^. Based on our screening results it is tempting to speculate that at least part of the anti-atherogenic effects upon FABP4 inhibition are conveyed through reduction of lipid accumulation in macrophages.

Much less is known about the two remaining newly identified regulators of macrophage LDL-uptake, *WBP5* (aka: *TCEAL9*) and *CMTM6*. *WBP5* encodes the transcription elongation factor A like 9, which binds polyproline ligands and contains a WW domain that mediates protein-protein interaction^32^. Interestingly, while knockdown of *WBP5* reduced DiI-LDL uptake in macrophages from both, CAD patients and healthy controls, this reduction was considerably more pronounced in patient cells, assuming a potential relevance for this gene in CAD pathogenesis. *CMTM6* is part of a little characterized chemokine-like factor gene superfamily. It is widely expressed across tissues, with high expression levels in monocytes^33^. Importantly, rs7640978 near *CMTM6* has been identified as associated with LDL-cholesterol and total cholesterol through the Global Lipids Genetics Consortium^34^. Our finding that reduction of *CMTM6* in macrophages reduces LDL-uptake adds support that *CMTM6* is the causal gene conferring the lipid association at this GWAS locus, and that inhibition of CMTM6 might be cardioprotective. Most interestingly, two very recent studies have identified CMTM6 as a binder and master regulator of cell-surface abundance of the immune checkpoint regulator PD-L1, expanding a putative therapeutic application for this gene to immuno-oncology^35,36^. Further studies will be required to elucidate the exact mechanisms how the newly-identified regulators impact macrophage biology.

With the aim to demonstrate the applicability of our platform towards an improved understanding of the molecular processes leading to foam cell formation, we further studied the role of the chemokine receptor CXCR4 and its ligands in this process. *CXCR4* has been described earlier as upregulated during macrophage foam cell formation^10,14^ and regulating clathrin-mediated endocytosis of acetylated LDL^37^. Consistent with a function during foam cell formation, we found here that knockdown of *CXCR4*, but not *CXCR7*, reduces LDL-uptake into monocyte-derived human macrophages under our settings. This observation is interesting since CXCR4 as well as the CXCR4 ligands CXCL12 and MIF have been implicated in atherogenesis and CAD: For CXCR4 itself, recent regression analyses have shown an association of the common *CXCR4* variant rs2322864, which *reduces* CXCR4 expression in carotid artery plaques, with *increased* risk for CAD in humans, and vascular CXCR4 was shown to reduce atherosclerosis by maintaining arterial integrity. However, it was also discussed that CXCR4 may also confer *pro-*atherogenic effects in cell types distinct from neutrophils, endothelial cells or smooth muscle cells^38^. *CXCL12* has been robustly associated through GWAS with CAD^39^, and increasing CXCL12 in *Apoe*^−/−^ mice reduced atherosclerosis^40^ and the foam cell content in lesions^40,41^. On the other hand, MIF showed a pro-atherogenic role in *Lldr*^−/−^ mice^42^. In our cell-based approach, knockdown of *MIF* strongly reduced, while exogenous addition of MIF stimulated DiI-LDL uptake into macrophages. Conversely, no such effect was found for either CXCL12 reduction nor addition under our experimental settings. The impact of MIF on LDL-uptake was dependent on CXCR4, since it was abrogated by either blocking the interaction of the two proteins via the compound AMD3100, or in *CXCR4* knock-down cells. Further studies will need to investigate how modulation of MIF/CXCR4 impacts macrophage LDL-uptake and the development of CAD *in vivo*. Further, while our initial experiments with pertussis toxin indicate that G-protein signaling might play a role, the exact mechanisms how the MIF/CXCR4 signaling axis modulates LDL-uptake will need to be further clarified. For instance, in addition to receptor-mediated processes, LDL may be internalized into cells through PI3K-dependent fluid-phase pinocytosis^43–45^. MIF induces PI3K signaling via CD74/CXCR4^46^, and the CXCL12/CXCR4 axis was previously shown to increase macropinocytosis of a cell-penetrating peptide in HeLa cells^47^. Combined, it is thus tempting to speculate that MIF/CXCR4/PI3K-signaling might confer macrophage LDL-uptake through pinocytosis, a hypothesis that should be followed in future studies.

Our study has several limitations. First, cell culture and *in vitro* differentiation from monocytes on siRNA-arrays may remain incomplete or introduce undesired changes to macrophages that might not be present *in vivo*. We have tried to control for that by monitoring for morphology as well as the expression of macrophage-specific markers, but only future applications with a larger range of assays will determine by which the model we newly introduce here matches or differs from alternative macrophage models. And second, we cannot exclude that the genes identified in our RNAi-screen impact LDL-uptake differently in alternative cell models, or influence lipid accumulation only in an indirect manner. While the gene set analyzed here had been highly prioritized for genes that are differentially regulated during foam cell formation, there could be interacting factors that might serve as the better therapeutic targets when manipulated by siRNAs or compounds. As we highlight at the case of *CXCR4*, unbiased analyses as they are now possible on our platform will help to substantiate existing and create new hypotheses on known and novel players of macrophage biology.

In summary, we introduce a scalable microscope-based platform for systematic functional studies in primary human monocyte-derived macrophages. We propose four genes as putative novel regulators of macrophage LDL-uptake that possibly directly may impact foam cell formation. Future studies will show whether the platform introduced here may assist diagnosis or risk assessment in patients, and whether the genes prioritized here through siRNA-screening have a therapeutic potential.

## Methods

### Selection of candidate genes and siRNAs

For selecting a gene set of 89 candidates, genes significantly up- and down-regulated during foam cell formation from human macrophages were selected from two previous publications^10,14^. Cho *et al*.^10^ report data acquired from two sets of experiments in which macrophages from healthy individuals were exposed to oxidized LDL (oxLDL) and either stimulated with MCSF or CXCL4. For both conditions, the 50 most strongly up- or downregulated genes were chosen for the current study. Hägg *et al*.^14^ report gene expression data from macrophages of 613 patients with CAD and 613 non-related controls. From this study, the 60 most strongly regulated genes in healthy individuals (up- or downregulation after oxLDL-stimulation) and the 30 most strongly regulated genes in cells derived from CAD patients were included. Few additional genes were chosen from further references. In order to increase the probability to identify possible targets for future CAD therapies, candidates from this selection were excluded from siRNA-screening if their described primary functions related to any of the following mechanisms: (1) primary structural protein and/or responsible for cell movement/attachment; (2) regulator of inflammatory response and/or part of anti-oxidative metabolism; (3) part of nucleic acid production, cell growth and/or proliferation; (4) ribosomal proteins; (5) pseudogene. A list of the 89 genes qualifying for microscope-based functional analyses is provided in Supplemental Table I.

Pre-designed 21-nt siRNAs were purchased from Ambion/Applied Biosystems (Waltham, MA, U.S.A.). To determine the best nucleotide sequence, all siRNA sequences suggested by Ambion were mapped to the human reference genome GRCh37 (Ensembl 66) using the software tool Bluegecko (J.K. Hériché, EMBL, unpublished). For the primary RNAi-screening, the three siRNAs targeting the largest number of transcripts were selected for each gene. For candidate genes qualifying for the replication screen, the two most significant siRNAs in the primary screen were analyzed.

### Study participants

For all experiments, approval from the institutional ethics committee was obtained in advance (IRB approval # S626-2011, University of Heidelberg). All methods were performed in accordance with their relevant guidelines and regulations. In both, primary and replication screen, a group of patients with coronary angiography-confirmed coronary artery disease was compared to age- and gender-matched healthy volunteers with normal coronary arteries on angiography. Males between 40 to 85 years of age were selected. Exclusion criteria were signs of infection, infectious and inflammatory diseases, cancer, kidney dysfunction, liver dysfunction, and neurological or psychiatric diseases, hemoglobin <10 g/dl, C-reactive protein >2 mg/dl, glomerular filtration rate <45 ml/min, and impaired left ventricular function. In the primary RNAi-screen, six patients and controls were studied, in the replication screen the number was increased to ten patients and ten controls. For details on the patient cohort participating in this study, see Supplemental Table II.

### Monocyte isolation and macrophage differentiation

After obtaining informed consent, 400 ml human peripheral blood per patient was obtained. Immediately after blood draw, PMBCs were isolated using Histopaque (FicoLite-H, Linaris, Dossenheim, Germany), followed by negative isolation with magnetic beads (EasySep^TM^ Human Monocyte Enrichment Kit, Stem Cell Technologies, Vancouver, Canada). After red blood cell lysis with distillated water and wash steps with 1 mM EDTA to reduce platelet and red blood cell contamination, monocytes were cultured in macrophage serum-free medium (Macrophage SFM (1×), liquid, Invitrogen, Waltham, MA, U.S.A.) supplemented with Nutridoma SP (Roche, Indianapolis, IN, USA) in a cholesterol-depleted environment without the addition of bovine serum at 37 °C/5% CO_2_. Following established protocols^10,11^, differentiation was induced by addition of 100 ng/ml recombinant human MCSF (Peprotech, Rocky Hill, NJ, U.S.A.) for three days. Differentiation of macrophages was confirmed by immunofluorescence staining for CD68 (CD68 Purified Goat poly-clonal IgG and polyclonal rabbit anti-goat IgG-FITC, Santa Cruz Biotechnology Inc., Dallas, TX, U.S.A.), after permeabilization with 0.1% Triton X (Sigma Aldrich, St. Louis, MO, U.S.A.).

### Reverse siRNA transfection

96- (High Content Imaging Glass Bottom Microplates, Corning, New York, NY, U.S.A.) and 384-well plates (BD Falcon 384 well, 120 µl Assay plates, San Jose, CA, U.S.A.) coated with siRNAs for solid phase reverse-transfection of cells were produced at the Advanced Biological Screening Facility, Bioquant, University of Heidelberg, as described previously^9^. After isolation, monocytes were distributed onto the prepared 96- or 384-well plates containing the dried transfection mix including the respective siRNA, Lipofectamine (Invitrogen, Waltham, MA, U.S.A.) and the adhesion factor fibronectine (human, Sigma-Aldrich, St. Louis, MO, U.S.A.).

Using a multi-channel pipette monocytes were seeded at a density of 25,000 cells/siRNA-coated 384-well for LDL- or oxLDL-uptake assays, and 20,000 cells/siRNA-coated 384-well for free cholesterol (FC) staining with Filipin or lipid droplet staining with Oil-Red(O). For siRNA-mediated down-regulation of regulators in the CXCR4 axis, macrophages were seeded at a density of 100,000/well. For CXCL12/MIF stimulation experiments, cells were seeded at a density of 25,000 cells/well.

siRNA uptake efficiency was tested by the use of Cy3-tagged control siRNA. A high percentage of Cy3-positive relative to Cy3-negative cells was in line with earlier findings from other cell lines and primary cells that showed that the solid-phase transfection protocol applied yields consistently high transfection rates^9,48^. Generally, the marginal wells of a 96- or 384-plate were not used because of plate effect errors in the marginal area. Non-silencing scrambled control siRNA was used as negative controls. The position of the siRNAs on the plate was randomly distributed across plates, and position was reshuffled for the replication screen. The following siRNAs were used as positive controls: *CSF1R*-siRNA (s3596), *LDLR*-siRNA (s237197), and *CD36*-siRNA (s2647) (all from Ambion, Waltham, MA, U.S.A.).

### Functional assays

For analyzing DiI-LDL-uptake in primary human monocyte-derived macrophages we followed a protocol we had established earlier for immortalized cell lines^12,13^. In brief, cell culture medium was replaced by culture medium containing 1% HPCD ((2-Hydroxy)-β-cyclodextrin, Sigma-Aldrich, St. Louis, MO, U.S.A.) and cells were left at 37 °C/5%CO_2_ for 45 min. After washing cells with imaging solution (0.2% BSA (Albumin from bovine serum, Sigma-Aldrich, St. Louis, MO, U.S.A.) in MEM without phenol red, containing 30 mM HEPES and 0.5 g/l NaHCO_3_ [pH 7.4] (Invitrogen, Waltham, MA, U.S.A.), 50 µg/ml DiI-LDL (Low Density Lipoprotein from Human Plasma, DiI complex, Invitrogen, Waltham, MA, U.S.A.) were added for 30 min at 4 °C and 20 min at 37 °C, followed by washing steps with imaging solution [pH 7.4], imaging solution [pH 3.5] and PBS, fixation with 3% Paraformaldehyde (Sigma-Aldrich, St.Louis, MO, U.S.A.), and counterstaining of nuclei and cytoplasm with Hoechst stain (1:1000, Hoechst Stain solution, Sigma-Aldrich, St.Louis, MO, U.S.A.) and deep red (1:20,000, CellMark^TM^ Deep Red Plasma membrane Stain, Invitrogen, Waltham, MA, U.S.A.). Identical steps were followed for analyzing DiI-oxLDL-uptake. For estimating free cellular cholesterol, after washing steps with cold PBS, cells were fixed with 3% PFA, stained with Filipin (1:20, Filipin III from Streptomyces filipinensis, Sigma-Aldrich, St. Louis, MO, U.S.A.) and DRAQ5 (1:500, BioStatus limited, Shepshed, Loughborough, United Kingdom) for 30 minutes. Similar steps were followed when staining cells with Oil-Red O (Sigma-Aldrich, St.Louis, MO, U.S.A.), for which cells were counterstained with Hoechst.

CXCL12 (Peprotech, Rocky Hill, CT, U.S.A.) was used at a concentration of 1 µg/ml, MIF (provided by Prof. Jürgen Bernhagen, RWTH Aachen) was used at a concentration of 1 µg/ml. Macrophages were obtained (25,000 cells/well) and incubated with BSA, CXCL12 or MIF for 8 or 24 hours. AMD3100 (Sigma-Aldrich, St. Louis, MO, U.S.A.) was used as an inhibitor of ligand-binding to CXCR4. For inhibition of GPCRs, differentiated macrophages were incubated with pertussis toxin (250 ng/ml; 516560 Pertussis Toxin, Merck Millipore, Billerica, MA, U.S.A.) alone, MIF alone and in combination with MIF, or with BSA (negative controls) for 24 hours.

### Automated image acquisition and analysis

Image acquisition was performed as described previously using an Olympus IX81 automated epifluorescence wide-field microscope and a 40× objective^12,13^. From each well, 28 non-overlapping images were taken at different positions within the well. Automated image analysis was performed using the software DetecTIFF, which uses an iteration algorithm to calculate signal intensity from multiple fluorescence channels at a single cell level^49^. Prior to analysis, all images were visually assessed for quality, and out-of-focus images as well as images with a minimal number of cells (as reflected by a median signal intensity value of zero or below) were excluded.

### Statistical analysis

Analysis of DiI-LDL fluorescence intensity per cell followed routines we have described earlier^12,13,49,50^. For the current study, the open-source program KNIME^51^ was used to establish an automated workflow for statistical analyses (see Supplemental Table III for analysis steps). In brief, from each image, DiI-LDL fluorescence intensity was quantified per cell, and intensities of all cells per image were averaged. For each well, the median intensity of all (up to 28) images per well was determined (Fig. SIII-I). For each technical replicate (i.e. plate), all intensities were normalized to the mean median intensity per well across all images from negative control wells (Fig. SIII-II). For siRNAs that had been analyzed in several wells of the plate, first the mean was calculated for each technical replicate (i.e. per plate/study participant). In a second step, these values were used to calculate the mean for the biological replicate for this siRNA (i.e., across multiple plates/participants) (Fig. SIII-III).

For statistical analysis, we further determined Deviation values and z-scores as commonly used in siRNA screening (Fig. SIII-IV)^12,13,48,50^. To counteract possible plate effects and positional effects, KNIME was programmed to calculate B-scores, reflecting a potentially more robust version of Z-scores^52–54^. A gene was considered a hit in the primary RNAi-screening, when in either the healthy control group, or the CAD patient group, or both, at least one siRNA for that gene had a Deviation value of >+1 or <−1, and another siRNA for that gene had a deviation of >+0.5 or <−0.5. The median of the deviation was calculated for each siRNA for control individuals (Md(C)), CAD patients (Md(P)) and the entire group comprising control individuals and CAD patients (Md(C + P)). In the replication screen, hit genes were defined when the deviation was >+1 or <−1 for both siRNAs tested. For the CXCR4 axis experiments, an unpaired two-tailed t-test was used followed by a post hoc Dunn’s multiple comparison test to calculate significance.

## Electronic supplementary material

Supplementary Information

Supplementary Datasheets
