# Supplementary material for: Systematic RNA-interference in primary human monocyte-derived macrophages: A high-throughput platform to study foam cell formation
Source: Sci Rep. 2018 Jul 12;8:10516. doi: 10.1038/s41598-018-28790-3 (PMC6043567; doi:10.1038/s41598-018-28790-3)
Supplement: Supplementary file 1 — Supplementary Information [file 41598_2018_28790_MOESM1_ESM.pdf]

SUPPLEMENTARY INFORMATION

to:

Systematic RNA-interference in primary human monocyte-derived macrophages: A high-throughput platform to study foam cell formation

Gabriele Domschke[a,b], Fabian Linden[b], Lukas Pawig[c], Anna Hafner[b], Mohammadreza Akhavanpoor[b], Jürgen Reymann[d], Andreas O. Doesch[b], Christian Erbel[b], Christian Weber[e,f], Hugo A. Katus[b], Heidi Noels[c], Holger Erfle[d,*], Christian A. Gleissner[b,*], Heiko Runz[a,g,*]

Affiliations:

- a Institute of Human Genetics, University of Heidelberg, 69120 Heidelberg, Germany;
- b Department of Cardiology, University of Heidelberg, 69120 Heidelberg, Germany DZHK (German Centre for Cardiovascular Research), Partner Site Heidelberg, 69120 Heidelberg, Germany;
- c Institute for Molecular Cardiovascular Research, RWTH Aachen University, University Clinic, 52074 Aachen, Germany;
- d BioQuant, University of Heidelberg, 69120 Heidelberg, Germany;
- e Institute for Cardiovascular Prevention, LMU Munich and German Centre for Cardiovascular Research (DZHK), partner site Munich Heart Alliance, Munich, Germany
- f Cardiovascular Research Institute Maastricht (CARIM), Maastricht University, Maastricht, the Netherlands
- g Present address: Biogen, Inc., Cambridge, MA, USA.

* these authors contributed equally

Correspondence:

Christian Gleissner, MD (christian.gleissner@med.uni-heidelberg.de) and
Heiko Runz, MD (heiko.runz@gmail.com)

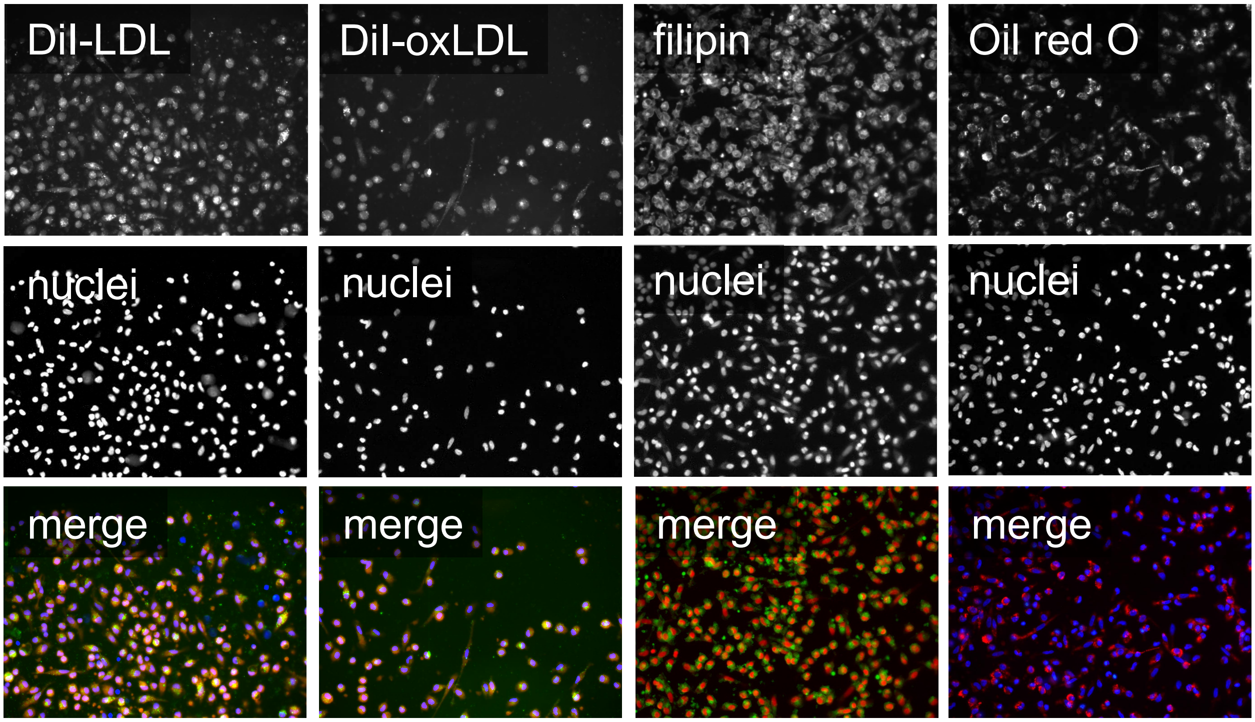

Supplemental Figure I. Automated microscopy to study lipid metabolism in cultured primary human monocyte-derived macrophages. Representative widefield microscopic images acquired on an automated high-throughput microscopy platform of macrophages analyzed for cellular uptake of DiI-LDL (left column), DiI-oxLDL (2nd column), cellular cholesterol content using filipin (3rd column), or lipid droplets using Oil-red O (right column).

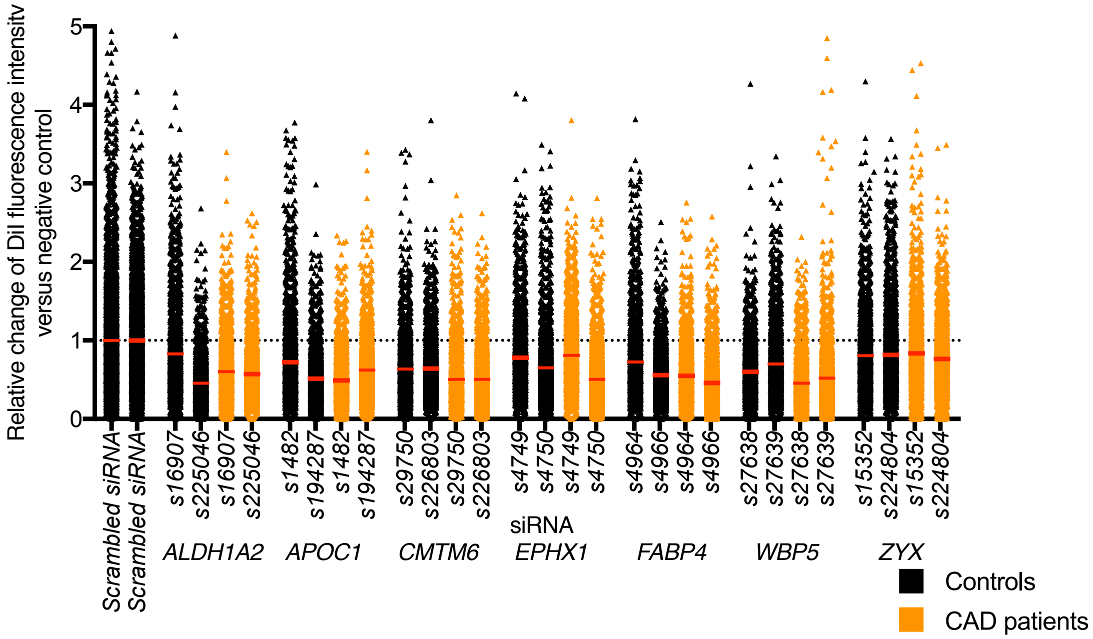

Supplemental Figure II. Relative change in DiI-LDL fluorescence intensities for seven candidate genes relative to control siRNA-treated cells. Each dot indicates the mean DiI fluorescence intensity from cell masks of one individual image frame. For each candidate gene nominated as putative foam-cell regulator during the primary screen, two siRNAs were spotted each on three wells of a 384 well plate. Shown are results from cells of 10 healthy individuals (black) and 10 patients with coronary artery disease (orange) as obtained during the replication screen with 3 technical replicates per individual. Intensity values were normalized to the mean of all negative controls wells.

I **Calculation of mean median intensity**

DiI-LDL signal from one image → median intensity

↓ n images / well

mean median intensity

II **Normalization for one plate**

DiI-LDL signal from one image → $\frac{median\ intensity_{candidate}}{mean\ median\ intensity_{ctr}}$

III **Calculation of mean for a specific siRNA**

$$mean_{siRNA\_plate} = \frac{normalized\ median\ intensity_{well\ 1} + ... + normalized\ median\ intensity_{well\ n}}{n\ wells}$$

↓

$$mean_{siRNA} = \frac{mean_{siRNA\_plate1} + ... + mean_{siRNA\_plate\ n}}{n\ plates}$$

IV **Calculation of Deviation or Z score**

$$Deviation = \frac{mean_{ctr} - mean_{cand}}{2 \times SEM_{cand}} \qquad SEM_{cand} = \frac{STDEV}{\sqrt{n_{techrep} - 1}} \qquad z - score = \frac{mean_{cand} - mean_{ctr}}{STDEV_{ctr}} \qquad z - score \pm 3 \times STDEV_{ctr}$$

Supplemental Figure III. Visualization of Data Analysis Strategy. For details, see Methods.

SUPPLEMENTAL TABLES

Table S1

Gene	Ensembl ID	Hagg et al. [ref. 13] healthy	Hagg et al. [ref. 13] CAD	Cho et al. [ref. 14] MCSF/oxLDL	Cho et al. [ref. 14] CXCL4/oxLDL	other reference
**ACTR3**	ENSG00000115091		x			
**PLIN2**	ENSG00000147872	x	x	x	x	
**AKR1B1**	ENSG00000085662	x			x	
**ALDH1A2**	ENSG00000128918				x	
**ANXA1**	ENSG00000135046	x			x	
**APOC1**	ENSG00000130208	x	x	x	x	
**APOL2**	ENSG00000128335				x	
**AQP9**	ENSG00000103569	x			x	
**ATP6V0E**	ENSG00000113732		x	x	x	
**BACH1**	ENSG00000156273				x	
**BASP1**	ENSG00000176788					x
**BATF**	ENSG00000156127		x	x	x	
**BIRC3**	ENSG00000023445		x	x	x	
**BSCL2**	ENSG00000168000		x	x	x	
**C14orf1**	ENSG00000133935	x	x	x	x	
**CALR**	ENSG00000179218		x	x	x	
**CAP2**	ENSG00000112186	x				
**CCL1**	ENSG00000108702		x	x	x	
**CCL22**	ENSG00000102962	x			x	
**CCPG1**	ENSG00000214882		x	x	x	
**CDKN2B**	ENSG00000147883					x
**CMTM6**	ENSG00000091317	x				
**CLIC1**	ENSG00000213719					x
**CORO1A**	ENSG00000102879	x	x	x	x	
**COX5B**	ENSG00000135940		x	x	x	
**CRABP2**	ENSG00000143320	x	x	x	x	
**CRIP1**	ENSG00000213145	x	x	x	x	
**DBI**	ENSG00000155368	x				
**DHRS9**	ENSG00000073737		x	x	x	
**DPP7**	ENSG00000176978	x				
**DUSP4**	ENSG00000120875				x	
**EPHX1**	ENSG00000143819	x	x	x	x	
**FABP4**	ENSG00000170323		x	x	x	
**FADS1**	ENSG00000149485	x	x	x	x	
**FADS2**	ENSG00000134824	x	x	x	x	
**FASN**	ENSG00000169710		x	x	x	
**SLC48A1**	ENSG00000211584					x
**GLT25D1**	ENSG00000130309					x
**IGFLR1**	ENSG00000126246				x	x
**GDF15**	ENSG00000130513		x	x	x	
**GLRX**	ENSG00000173221	x			x	
**GPX4**	ENSG00000167468				x	
**H3F3A**	ENSG00000163041		x	x	x	
**HEBP1**	ENSG00000013583	x			x	
**HMOX1**	ENSG00000100292		x	x	x	
**HNRPM**	ENSG00000099783				x	
**HPCAL1**	ENSG00000115756	x			x	
**KRR1**	ENSG00000111615	x			x	
**ACOT13**	ENSG00000112304					x
**RNF19B**	ENSG00000116514					x
**ID2**	ENSG00000115738	x				

**IFITM1**	ENSG00000185885		x	x	x	
**ZNF706**	ENSG00000120963					
**LPL**	ENSG00000175445	x			x	
**LRMP**	ENSG00000118308				x	
**TMEM97**	ENSG00000109084	x	x	x	x	
**MGLL**	ENSG00000074416	x				
**MORF4L1**	ENSG00000185787					x
**MRPS2**	ENSG00000122140					x
**MYB**	ENSG00000118513		x	x	x	
**IL32**	ENSG00000008517		x	x	x	
**PLAUR**	ENSG00000011422	x				
**PLEK**	ENSG00000115956	x				
**PPAP2B**	ENSG00000162407	x			x	
**PSIP1**	ENSG00000164985	x			x	
**RAP2B**	ENSG00000181467				x	
**C13orf15**	ENSG00000102760					x
**RRAGD**	ENSG00000025039				x	
**S100A6**	ENSG00000197956	x				
**SAT1**	ENSG00000130066				x	
**SCD**	ENSG00000099194	x			x	
**SH2D1A**	ENSG00000183918				x	
**SMARCA4**	ENSG00000127616					x
**SMPDL3A**	ENSG00000172594		x	x		
**SNF8**	ENSG00000159210					x
**SQLE**	ENSG00000104549	x	x	x	x	
**SQSTM1**	ENSG00000161011	x	x	x	x	
**TALDO1**	ENSG00000177156	x			x	
**TNFRSF21**	ENSG00000146072		x	x	x	
**TPT1**	ENSG00000133112		x	x	x	
**TXN**	ENSG00000136810	x	x	x	x	
**GLRX3**	ENSG00000108010		x	x	x	
**UCHL1**	ENSG00000154277		x	x	x	
**VAT1**	ENSG00000108828				x	
**WBP5**	ENSG00000185222		x	x	x	
**ZBED2**	ENSG00000177494				x	
**ZNF259**	ENSG00000109917					x
**ZYX**	ENSG00000159840	x				
**LGALS3BP**	ENSG00000108679					x

Supplemental Table I. 89 genes regulated during foam cell formation functionally analyzed for an impact on cellular DiI-LDL uptake during this study. Genes were selected based on their differential mRNA expression based on references [10,13].

Table S2

Parameter	Total	Both screens combined			Primary screen			Replication Screen		
		Control	CAD	p	Control	CAD	p	Control	CAD	p
**n**	**32**	16	16		6	6		10	10	
**Age (years)**	**60±9**	56±7	65±9	0.003	55±8	63±10	0.240	56±7	66±8	0.007
**CAD**	**16 (50%)**	-	-	-	-	-	-	-	-	-
**Hypertension**	**19 (59%)**	4 (25%)	11 (69%)	<0.001	2 (33%)	5 (84%)	0.102	2 (20%)	10 (100%)	<0.001
**Hyperlipidemia**	**19 (59%)**	8 (50%)	11 (69%)	0.134	5 (83%)	5 (84%)	0.102	3 (30%)	6 (60%)	0.527
**Diabetes mellitus**	**16 (50%)**	2 (13%)	3 (19%)	0.012	0 (0%)	2 (33%)	0.414	2 (20%)	1 (10%)	0.011
**Family history**	**14 (44%)**	5 (31%)	9 (56%)	0.617	3 (50%)	3 (50%)	1.000	2 (20%)	6 (60%)	0.527
**Smoking history**	**16 (50%)**	9 (56%)	7 (44%)	0.617	1 (17%)	5 (84%)	0.102	8 (80%)	2 (20%)	0.0.58
**MI**	**3 (9%)**	0 (0%)	3 (19%)	0.012	0 (0%)	3 (50%)	<0.001	0 (0%)	0 (0%)	1.000
**PCI**	**15 (47%)**	0 (0%)	15 (94%)	<0.001	0 (0%)	6 (100%)	<0.001	0 (0%)	9 (90%)	0.011
**CABG**	**3 (9%)**	0 (0%)	3 (19%)	0.012	0 (0%)	1 (17%)	0.102	0 (0%)	2 (20%)	0.058
**Peripheral artery disease**	**2 (6%)**	0 (0%)	2 (13%)	0.003	0 (0%)	1 (17%)	0.102	0 (0%)	1 (10%)	0.011
**Stroke, TIA**	**1 (3%)**	0 (0%)	1 (6%)	<0.001	0 (0%)	1 (17%)	0.102	0 (0%)	0 (0%)	1.000
**Total cholesterol (mg/dl)**	**183±42**	195±49	171±29	0.138	147±23	164±23	0.240	224±34	175±33	0.005
**HDL (mg/dl)**	**44±15**	44±15	44±16	0.809	34±16	40±21	0.310	50±11	47±14	0.353
**LDL (mg/dl)**	**109±29**	121±30	98±25	0.047	94±13	95±11	0.937	138±25	99±31	0.011
**Triglycerides (mg/dl)**	**149±74**	154±77	144±73	0.809	97±45	138±87	1000	188±73	148±69	0.247

Supplemental Table II. Clinical and demographic characteristics of patients analyzed in this study. Control individuals and coronary artery disease (CAD) patients included in primary and replication screens are shown. Means ± standard deviations or absolute numbers (percentages) are indicated. P values were calculated by Mann-Whitney test (continuous variables) or Chi square test (categorical variables). N, sample number; MI, myocardial infarction; PCI, percutaneous coronary intervention; CABG, coronary artery bypass grafting; TIA, transient ischemic attack; HDL, high-density lipoprotein; LDL, low-density lipoprotein.

SUPPLEMENTAL DATASHEETS

see accompanying file: [Domschke_SupplDatasheets]

Supplemental Datasheet 1
Results of primary RNAi-screening for functional regulators of DiI-LDL uptake into cultured primary human macrophages among 89 foam-cell regulated genes
The impact on DiI-LDL uptake upon candidate gene knockdown was analyzed with 3 independent siRNAs/gene. SiRNAs meeting significance criteria (z-score/Deviation >|1|) are highlighted in red (increased LDL-uptake relative to control-siRNA treated cells) or green (reduced LDL-uptake relative to control-siRNA treated cells) as mean medians of deviations (Md). Negative values indicate reduction in DiI-LDL uptake. C, control individuals; P, coronary-artery disease patients. Column V [Md(C+P)] indicates means across patients and control individuals in this study. Column W [Md(C)-Md(P)] indicates difference in means between patients and controls in this study. *** met criteria for inclusion in validation screening; NA, not available.

Supplementary Datasheet 2
Results of validation RNAi-screening for functional regulators of DiI-LDL uptake into cultured primary human macrophages among 7 putative foam-cell regulators identified in primary screen
For validation of primary screen results, the impact on DiI-LDL uptake upon knockdown of seven selected candidate genes was analyzed with 2 independent siRNAs/gene. SiRNAs meeting significance criteria (z-score/Deviation >|1|) are highlighted in green (reduced LDL-uptake relative to control-siRNA treated cells) as mean medians of deviations (Md). Negative values indicate reduction in DiI-LDL uptake. C, control individuals; P, coronary-artery disease patients. Columns AE and AI [Md(C+P)] indicate means across patients and control individuals in this study. Columns AF and AJ [Md(C)-Md(P)] indicate difference in means between patients and controls in this study. siRNAs with Md(C)-Md(P) >|1| are highligthed in red. Columns AG-AJ indicate meas for all measurements per siRNA during replication screen. Genes with ³2 significant siRNAs validated in replication screen are highlighted by **.; NA, not available.
